# Antibacterial efficacy of titanium-containing alloy with silver-nanoparticles enriched diamond-like carbon coatings

**DOI:** 10.1186/s13568-015-0162-z

**Published:** 2015-12-09

**Authors:** Norbert Harrasser, Sebastian Jüssen, Ingo J. Banke, Ralf Kmeth, Ruediger von Eisenhart-Rothe, Bernd Stritzker, Hans Gollwitzer, Rainer Burgkart

**Affiliations:** Clinic of Orthopedics and Sports Orthopedics, Klinikum rechts der Isar, Technical University of Munich, Ismaninger Str. 22, 81675 Munich, Germany; Experimental Physics IV, Institut für Physik, Augsburg University, Universitätsstr. 1, 86135 Augsburg, Germany; ATOS Clinic, Effnerstr.38, 81925 Munich, Germany

**Keywords:** Implant-associated infections, Diamond-like carbon, Silver, Titanium, Antibacterial coating

## Abstract

Silver ions (Ag^+^) have strong bactericidal effects and Ag-coated medical devices proved their effectiveness in reducing infections in revision total joint arthroplasty. We quantitatively determined the antimicrobial potency of different surface treatments on a titanium alloy (Ti), which had been conversed to diamond-like carbon (DLC-Ti) and doped with high (Ag:PVP = 1:2) and low (Ag:PVP = 1:10 and 1:20) concentrations of Ag (Ag-DLC-Ti) with a modified technique of ion implantation. Bacterial adhesion and planktonic growth of clinically relevant bacterial strains (*Staphylococcus epidermidis,Staphylococcus aureus, and Pseudomonas aeruginosa*) on Ag-DLC-Ti were compared to untreated Ti by quantification of colony forming units on the adherent surface and in the growth medium as well as semiquantitatively by determining the grade of biofilm formation by scanning electron microscopy. (1) A significant (p < 0.05) antimicrobial effect could be found for all Ag-DLC-Ti samples (reduced growth by 5.6–2.5 logarithmic levels). (2) The antimicrobial effect was depending on the tested bacterial strain (most for *P. aeruginosa*, least for *S. aureus*). (3) Antimicrobial potency was positively correlated with Ag concentrations. (4) Biofilm formation was decreased by Ag-DLC-Ti surfaces. This study revealed potent antibacterial effects of Ag-DLC-Ti. This may serve as a promising novel approach to close the gap in antimicrobial protection of musculoskeletal implants.

## Introduction

With a constantly rising demand for orthopedic surgery with approximately 2.6 million orthopedic implants performed annually in the United States, the frequency of associated infections is bound to increase (Liu et al. 2012; Zimmerli and Ochsner 2003; Kurtz et al. 2008). Prevention of periprosthetic joint infections (PJI) has therefore an important impact not only on patient morbidity but also on the cost effectiveness of hospital care (Gosheger et al. 2004). Management of PJI often requires multiple staged surgeries and the use of antibiotics as a supportive therapy (Giulieri et al. 2004; Zimmerli and Ochsner 2003). A major problem in septic revision surgery is the formation of biofilm on implanted foreign materials (Schrenzel et al. 2004). These biofilms contain 5–50 µm thick glycoprotein matrices that protect the bacteria through a diffusion limitation process, and increase their resistance to antibodies, macrophages, and antibiotics (Ceri et al. 1999). Once a significant amount of biofilm has formed, eradication of infection is nearly impossible without removing the implant (Zimmerli and Ochsner 2003).

In order to decrease the infection and especially reinfection rate several methods have been developed, at which rising occurrences of antibiotic resistances among bacteria make antibiotic-based strategies more and more questionable (Jamsen et al. 2010; Schmidmaier et al. 2006; Hetrick and Schoenfisch 2006; Poelstra et al. 2000). In this context alternative methods are favored. Promising could be the use of non-antibiotic “active” antibacterial coatings which release antibacterial agents, e.g. silver ions (Ag^+^) (Gosheger et al. 2004; Hardes et al. 2007; Harrasser et al. 2015), copper ions (Cu^++^) (Shirai et al. 2009; Baena et al. 2006), nitric oxide (Holt et al. 2011; Nablo et al. 2005), chlorhexidine/chloroxylenol (Darouiche et al. 1998) or chitosan (Bumgardner et al. 2003). Compared to antibiotics these agents act more broadly against a wide range of bacteria. In addition, at least proven for the use of silver (Ag), microbes without intrinsic resistance cannot gain resistance (Lee et al. 2005). Information on the use of these bactericidal coatings on wear surfaces and direct bone contact is lacking since Ag-coatings have been used so far only on surfaces without direct bone or joint contact. This fact is important, given that, e.g. in total knee replacement roughly 50 % of the surface is exposed to synovial fluid and in main parts tribologically active. On the other hand, revision prostheses are usually composed of large stems applied intramedullary which additionally represent a vulnerable surface area for bacterial contamination. To summarize, in septic revision surgery a relevant portion of the susceptible prosthesis is not protected against bacterial reinfection. Antibacterial-agent-enriched diamond-like carbon (DLC) coatings may solve this dilemma. The term DLC is used to describe hydrogen-free carbon solids that contain an amorphous network of tetrahedrally and trigonally hybridized carbon atoms with physical properties tending to be intermediate between those of graphite and those of diamond (Dearnaley 1993). DLC is an ideal surface coating for prosthetic joints, because it is wear resistant, atomically smooth, and corrosion resistant, has a low friction coefficient, and is immune to scratching by third body wear particles (Morrison et al. 2006). Therefore, if DLC coating is applied on titanium (Ti), a material not used for wear surfaces due to its high sensitivity to contact wear and fretting corrosion, it improves its wear resistance and makes it suitable for tribologically loaded joint parts (Firkins et al. 1998). Additionally, DLC coating can improve the osseointegration of titanium making it even more valuable for orthopedic applications (Mändl et al. 2001). Another property of this coating is the ability to use it as a carrier for ions, e.g. Ag^+^. By release of Ag^+^, DLC coatings on Ti can act as local antibacterial agents, and therefore extend its medical implications to septic revision surgery (Cloutier et al. 2014; Katsikogianni et al. 2006; Dwivedi et al. 2013). DLC coatings are not without limitations since structural properties can vary widely depending on the deposition techniques employed. One of the greatest challenges of DLC technology is adhesion and internal stresses (Xu and Pruitt 1999). Several DLC technologies are described, at which DLC films usually are deposited on surfaces. The bonding strength between coating and surface seems to be the weakest point (Walter et al. 1997). If the DLC layers tend to dissolve from the surface positive tribological features can quickly deteriorate leading to high grade wear (Roy and Lee 2007). This could be conflicting if DLC coated Ti was used on wear surfaces. On the other hand, methods of DLC coating with strong linking to the sample surface are described (Liu et al. 2004). Thus, the risk of detachment of the DLC coating from the surface, even under shear forces is minimized (Schwarz and Stritzker 2010; Popa et al. 2013). In this context, among the techniques described for the synthesis of DLC coatings a modified method of plasma immersion ion implantation (PIII) has proven promising advantages (Schwarz and Stritzker 2010).

We have used a new technique to incorporate silver (Ag) DLC thin films on Ti samples (Ag-DLC-Ti) in order to provide these otherwise inert medical-devices with antimicrobial properties. Bactericidal potency is studied on the sample’s surface and the surrounding growth medium. This study provides valuable information for determining the suitability of Ag-DLC-Ti as antibacterial materials for septic revision implants.

## Methods

### Study substrates

We used cylindrical substrates (diameter: 10 mm, height: 2 mm) of corundum-blasted medical TiAl6V4 (Ra ~ 5 µm; Goodfellow GmbH, Nauheim, Germany). The samples were coated with Ag-doped DLC, incubated for 24 h and afterwards tested for their antimicrobial effects on the surface (bacterial sessile growth) and the surrounding growth medium (bacterial planktonic growth).

DLC doping with Ag was carried out with polyvinylpyrrolidone (PVP) as a stabilizing agent. The first testing series was conducted with high concentrations of Ag within the DLC coating (Ag:PVP = 1:2) and three different bacterial strains (*Staphylococcus epidermidis, Staphylococcus aureus, Pseudomonas aeruginosa*). For reduction of potential toxic side-effects of Ag on eukaryotic cells a reduction of Ag^+^ would be advantageous (Hardes et al. 2007). Hence, detection of minimal necessary Ag^+^ concentration in DLC-Ti samples was evaluated in a further testing series with low concentrations of Ag within the DLC coating (Ag:PVP = 1:10 and 1:20). This series was only conducted with the most resistant strain against Ag from testing series one (*S. aureus*). Sample features and used bacterial strains are summarized in Table 1.

**Table 1:** Features of the different testing groups

Testing group	Ag:PVP	Bacterial strain
Ag-concentration of Ag-DLC-Ti: High	1:2	*Staphylococcus* *epidermidis* (ATCC35984) *Staphylococcus* *aureus* (ATCC25923) *Pseudomonas aeruginosa* (ATCC 27835)
Ag-concentration of Ag-DLC-Ti: Low	1:10 1:20	*Staphylococcus* *aureus* (ATCC25923)
SEM-evaluation	1:2	*Staphylococcus* *epidermidis* (RP62a)

### DLC film deposition

**Fig. 1:**
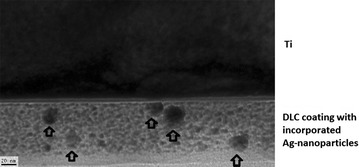
Transmission electron microscopy (TEM) image of silver nanoparticles of AG-DLC-Ti (note: major nanoparticles are marked with *arrows*)

DLC-processing of all plates was performed at the department of experimental physics IV (University of Augsburg, Germany) according to a modified technic of ion irradiation of polymers. The coating process is described fully elsewhere (Schwarz and Stritzker 2010). Briefly summarized, an ethanol-based solution of silver nitrate, benzoin and polyvinylpyrrolidone (PVP) was prepared and exposed to UV light to initiate photochemical reduction. This results in a colloidal solution of silver nanoparticles stabilized by PVP, with amount and size of the particles depending on the initial PVP-Ag and Ag-Benzoin ratios. The solution was then applied to the substrate via dip-coating. The resulting silver nanoparticle-containing polymer layer is subsequently transferred to amorphous carbon by plasma immersion ion implantation (PIII). A big advantage of this procedure is the fact that setting of amount and size of silver nanoparticles is independent from DLC-formation. In summary, the following surfaces have been investigated: Ti (untreated) = corundum-blasted medical TiAl6V4 alloy; DLC-Ti = corundum-blasted medical TiAl6V4 with DLC surface coating; Ag-DLC-Ti = corundum-blasted medical TiAl6V4 with DLC surface coating containing nanocolloidal silver in different molar ratios (high concentrated: Ag:PVP = 1:2; low concentrated: Ag:PVP = 1:10 and 1:20; Note: Amount of Ag^+^ is higher in 1:10 compared to 1:20). A TEM (transmission electron microscopy)-image of the Ag-DLC coating on Ti is given in Fig. 1.

### Sterilization of samples and sealing of uncoated surface with paraffin wax

**Fig. 2:**
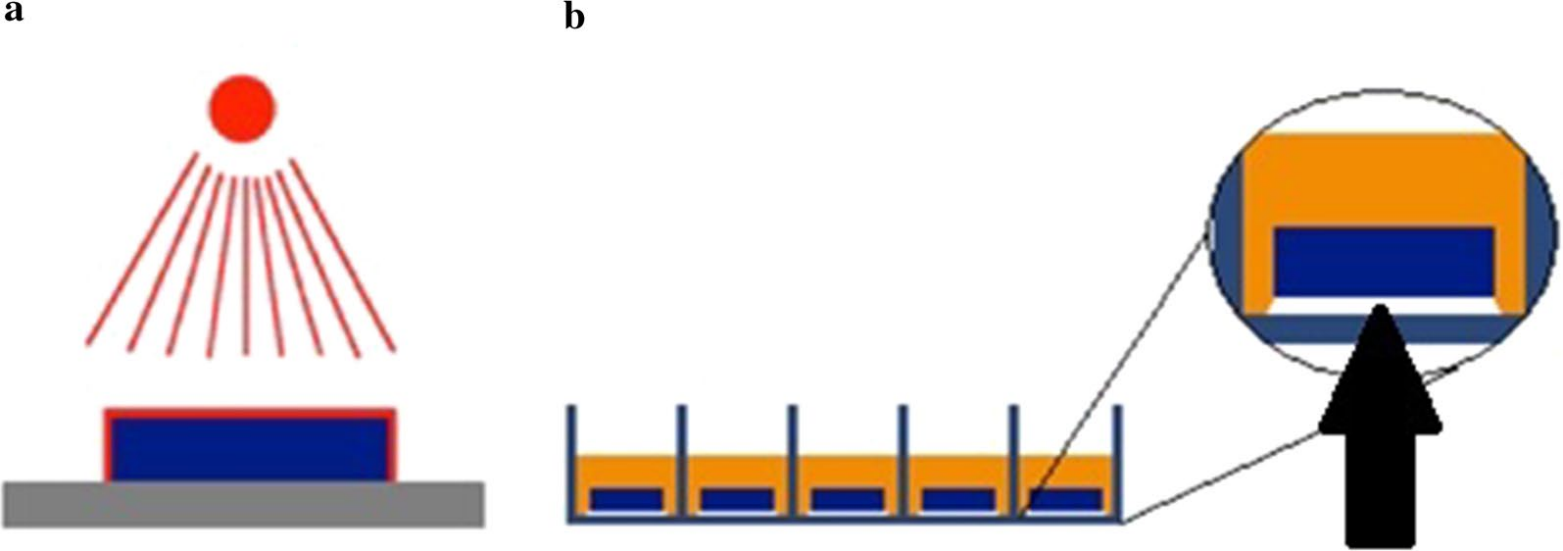
Sample preparation; ion irradiation of samples with missing irradiation of the sample’s lower surfaces (**a**), placement of samples in well culture plates with paraffin wax (*arrow*) covering the sample’s lower surface (**b**)

Samples were rinsed with distilled water for 10 min, air-dried in a laminar flow cabinet and thereafter sterilized with gamma-beam with the dose of 26.5 kGy (Isotron Deutschland GmbH, Allershausen, Germany). All manipulations of the samples were conducted by holding the lower surface. As a consequence these parts of the samples were not surface treated and needed protection from the testing environment. Hence, paraffin wax was first autoclaved in a glass container with 120 °C for 20 min (Varioklav^®^, H+P Labortechnik, H+P Labortechnik AG, Oberschleißheim, Germany), the samples’ lower surfaces were then dip-coated in the solvent paraffin wax so that a thin protection layer was formed. Specimens were then placed in 24-well culture plates (Fig. 2a, b). Pretesting with paraffin wax revealed no intrinsic antimicrobial potential and was therefore appropriate as a mechanical sample stabilizer.

### Bacterial strains and preparation of inocula

Bacterial strains (LGC Standards GmbH, Wesel, Germany) selected in the study for determination of surface and planktonic growth were the most common causative pathogens associated with PJI, namely *S. epidermidis* (ATCC35984), *S. aureus (*ATCC25923*)* and *P. aeruginosa (*ATCC 27835) (Zimmerli and Moser 2012; Darouiche et al. 1998; Zimmerli and Ochsner 2003). *S. epidermidis* (RP62a), as a strong slime producing variant, was used for SEM (scanning electron microscopy)-evaluation of biofilm formation on the samples. Test strains were routinely cultured in Columbia Agar with 5 % sheep blood (Becton–Dickinson, Heidelberg, Germany) at 37 °C overnight before testing. Bacteria were then harvested by centrifugation, rinsed, suspended, diluted in sterile phosphate buffered saline (PBS) and adjusted by densitometry to a MacFarland 0.5 standard (MacFarland Densimat™, BioMérieux, Marcy l’Etoile, France) up to a CFU count of 1 × 10^5^ CFU/ml. To control bacterial concentration, 100 μl of each suspension was again cultured for 24 h at 37 °C. After 24 h serial dilutions of this suspension were plated on Colombia-Agar. The colonies were counted and colony numbers calculated accordingly. For the study every suspension with its known bacterial concentration was diluted with DMEM + 10 % FCS to reach the targeted value for bacterial concentration (10^5^ CFU/ml). Sample plates with paraffin-coated lower surfaces were placed in 24-well culture plates and 1 ml of 10^5^ CFU/ml bacterial suspensions were added. Incubation of the well plates was conducted for 24 h at 37 °C.

### Analysis

Bacterial surface adhesion was evaluated by determining bacterial concentration on the specimen. Bacterial planktonic growth was measured in the growth medium. For every group four independent testing runs with four different samples were conducted. Therefore, altogether 16 samples were tested for every group.

### Determination of bacterial growth on sample surfaces

Colonized sample plates were removed from the wells with a sterile forceps, carefully rinsed twice with sterile PBS, transferred to vials containing 3 ml of sterile PBS and sonicated for 7 min (Elmasonic S60H, Elma, Singen, Germany) to remove adhering bacteria. 100 μl of the fluid were aspirated, plated on Colombia Agar at 37 °C for 24 h and quantified after incubation (CFU/ml).

SEM-analysis was conducted semiquantitatively to evaluate inhibition of biofilm formation. SEM-images were compiled of native Ti, DLC-Ti and Ag-DLC-Ti (Ag:PVP = 1:2). Biofilm formation was quantified in five categories: (1) no biofilm formation, (2) biofilm covering less than 25 % of the surface, (3) biofilm covering between 25 and 75 % of the surface, (4) biofilm covering more than 75 % of the surface, (5) biofilm formation covering the entire surface.

### Determination of bacterial planktonic growth

A 700-μl volume of each well was supplemented with 700 μl neutralizing solution as described by Tilton (Tilton and Rosenberg 1978) (1.0 g sodium thioglycolate + 1.46 g sodium thiosulfate in 1000 ml deionized water). The neutralizing solution acts as an inhibitor for reminiscent metal toxicity on bacteria. The suspension was plated on Columbia Agar after serial dilutions and incubated at 37 °C for 24 h. Thereafter, CFU were quantified and extrapolated to CFU/ml.

### Statistics

All results are presented as mean ± standard deviation. Statistical significance was computed using non-parametric methods and the method of closed testing procedure (Kruskal–Wallis and Mann–Whitney U test). P < 0.05 was considered statistically significant. Statistical tests were performed with use of SPSS (version 20.0; Chicago, Illinois). Statistical analysis was conducted per consultation with the Institute of Medical Statistics and Epidemiology (Klinikum rechts der Isar, Technische Universität München, Munich, Germany).

## Results

### Antimicrobial effect of high concentrated (Ag:PVP = 1:2) Ag-DLC-Ti on *S. epidermidis* ATCC35984, *S. aureus* ATCC25923 and *P. aeruginosa* ATCC27835

Average viable counts of bacteria recovered from the samples and in the supernatant growth medium are summarized in Table 2.

**Table 2:** Amount/concentration. changes (log-levels/%) and p values (<0.05: significant) of CFU of different bacterial strains on Ti samples with high and low concentrated Ag-DLC

High concentrated Ag-DLC-Ti	Ti (untreated)	DLC-Ti	Ag-DLC-Ti (Ag:PVP 1:2)
*S. epidermidis*			
*Surface adhesion*			
CFU ± SD	2.1 × 10^5^ ± 7.6 × 10^4^	3.3 × 10^5^ ± 2.4 × 10^5^	8.1 × 10^0^ ± 1.9 × 10^1^
Changes compared to Ti (log-levels/%)^a,b^		+0.2/+57 %	−4.4/−99.9 %
p values		0.149	<0.05
*Growth in surrounding medium*			
CFU/ml ± SD	4.5 × 10^5^ ± 2.8 × 10^5^	1.0 × 10^6^ ± 4.0 × 10^5^	3.4 × 10^2^ ± 1.1 × 10^3^
Changes compared to Ti (log-levels/%)^a,b^		+0.3/+122.2 %	−2.8/−99.9 %
p values		<0.05	<0.05
*S. aureus*			
*Surface adhesion*			
CFU ± SD	7.3 × 10^6^ ± 3.1 × 10^6^	7.8 × 10^6^ ± 2.4 × 10^6^	1.7 × 10^4^ ± 3.1 × 10^4^
Changes compared to Ti (log-levels/%)^a,b^		+0.03/+6.8 %	−2.6/−99.9 %
p values		0.428	<0.05
*Growth in surrounding medium*			
CFU/ml ± SD	1.1 × 10^8^ ± 4.1 × 10^7^	8.4 × 10^7^ ± 6.1 × 10^7^	3.6 × 10^5^ ± 5.4 × 10^5^
Changes compared to Ti (log-levels/%)^a,b^		−0.1/−23.6 %	−2.5/−95.9 %
p values		0.213	<0.05
*P. aeruginosa*			
*Surface adhesion*			
CFU ± SD	1.6 × 10^6^ ± 7.7 × 10^5^	1.3 × 10^6^ ± 5.7 × 10^5^	3.8 × 10^0^ ± 1.5 × 10^1^
Changes compared to Ti (log-levels/%)^a,b^		−0.09/−18.6 %	−5.6/−99.9 %
p values		0.533	<0.05
*Growth in surrounding medium*			
CFU/ml ± SD	2.4 × 10^8^ ± 3.8 × 10^7^	2.1 × 10^8^ ± 2.7 × 10^7^	7.4 × 10^2^ ± 1.6 × 10^3^
Changes compared to Ti (log-levels/%)^a,b^		−0.06/−12.5 %	−5.5/−99.9 %
p values		0.161	<0.05

Low concentrated Ag-DLC-Ti	Ti (untreated)	DLC-Ti	Ag-DLC-Ti	
			Ag:PVP 1:10	Ag:PVP 1:20
*S. aureus*				
*Surface adhesion*				
CFU ± SD	6.8 × 10^5^ ± 7.6 × 10^4^	9.7 × 10^5^ ± 2.4 × 10^4^	5.9 × 10^3^ ± 4.9 × 10^3^	1.3 × 10^4^ ± 5.2 × 10^3^
Changes compared to Ti (log-levels/%)^a,b^		+0.2/+57 %	−1.1/−91.8 %	−0.7/−81.9 %
p values		0.149	<0.05	<0.05
*Growth in surrounding medium*				
CFU/ml ± SD	3.1 × 10^5^ ± 1.8 × 10^5^	5.5 × 10^5^ ± 2.0 × 10^5^	5.2 × 10^4^ ± 3.8 × 10^4^	1.4 × 10^5^ ± 5.4 × 10^4^
Changes compared to Ti (log-levels/%)^a,b^		+0.3/+122.2 %	−0.8/−98.3 %	−0.3/−54.8 %
p values		<0.05	<0.05	<0.05

*CFU* colony forming units, *SD* standard deviation

^a^log-levels = bacterial counts calculated as shown in following equation: log-levels = log_10_(CFU of Ag-DLC-Ti) − log_10_(CFU of untreated Ti)

^b^Positive values (log-levels/%) express increased bacterial growth on DLC-Ti/Ag-DLC-Ti compared to untreated Ti, negative values express reduced bacterial growth on DLC-Ti/Ag-DLC-Ti compared to untreated Ti

**Fig. 3:**
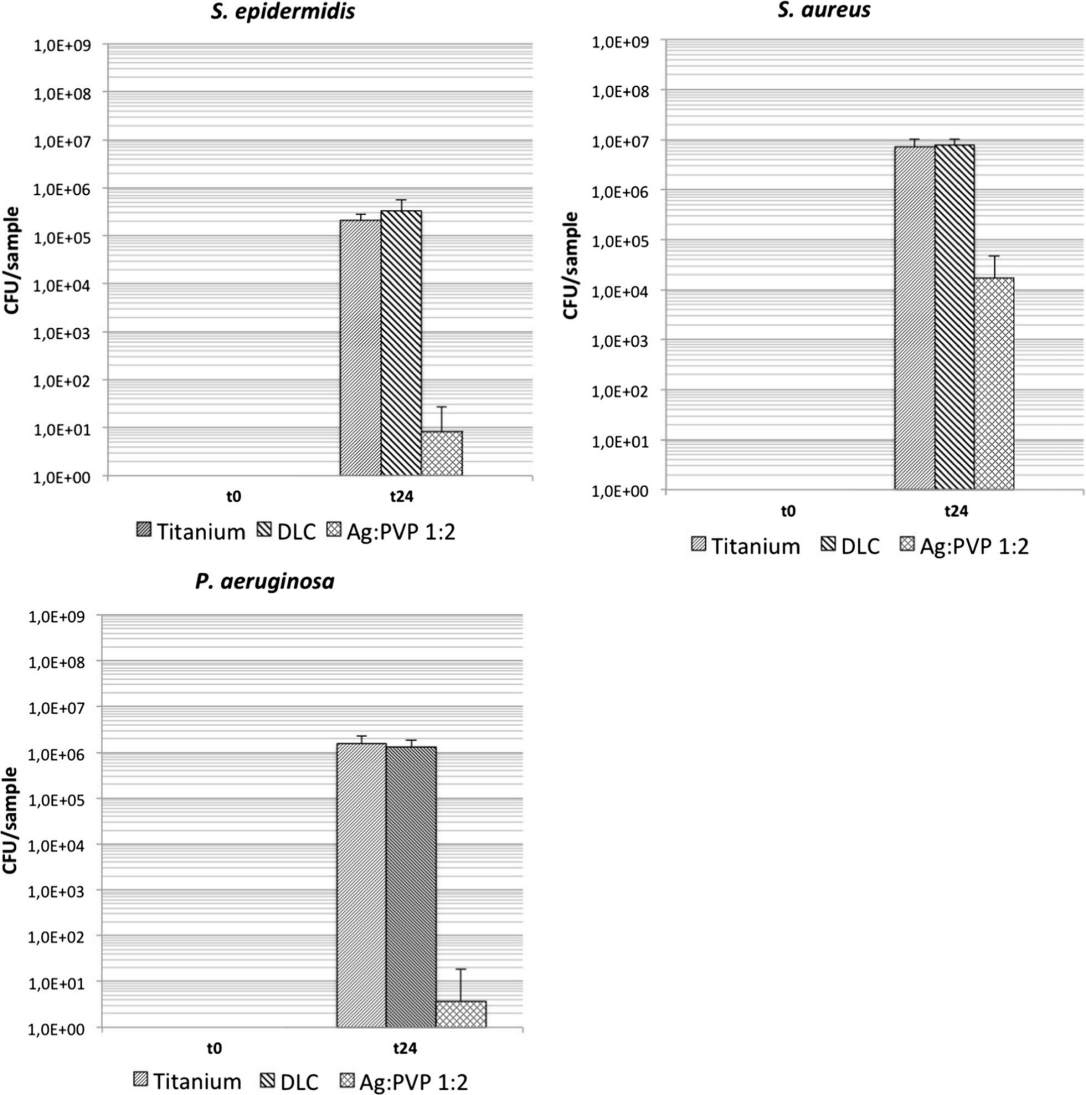
Bacterial growth of tested strains on the sample surfaces (Ti, DLC-Ti, and high concentrated Ag-DLC-Ti); t = 0: before incubation; t = 24 h: after incubation

Analysis of bacterial surface adhesion showed strain dependent differences in growth in the absence of Ag. On untreated Ti plates on average 2.1 × 10^5^ CFU of *S. epidermidis*, 7.3 × 10^6^ CFU of *S. aureus* and 1.6 × 10^6^ CFU of *P. aeruginosa* adhered after 24 h of incubation (Fig. 3). Compared to native plates, DLC-Ti samples showed not significant strain dependent changes of bacterial amounts. On all Ag-DLC-Ti surfaces (Ag:PVP = 1:2), compared to native plates, a significant reduction of bacterial amount was detected.

**Fig. 4:**
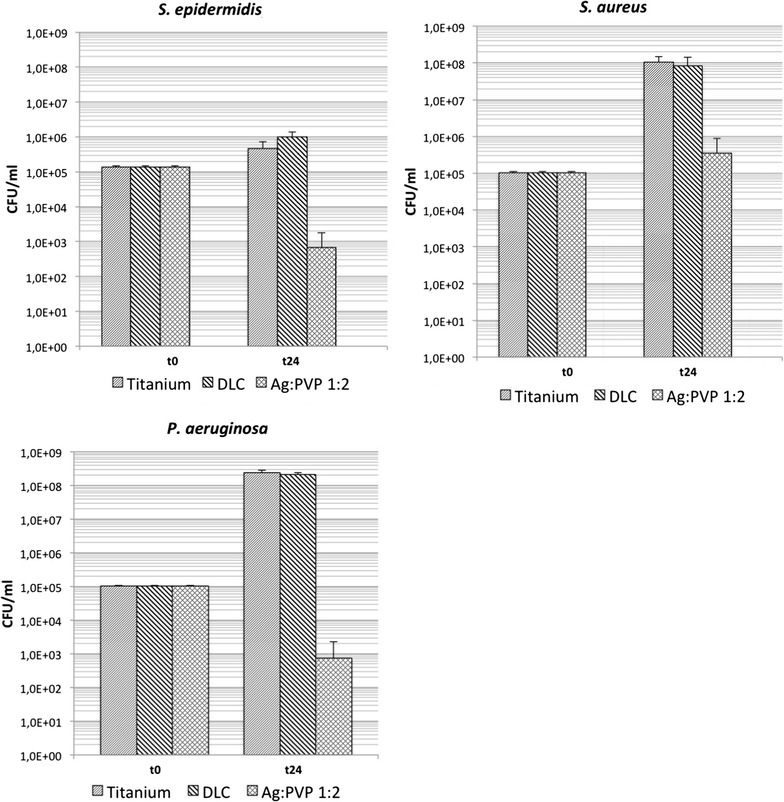
Bacterial growth of tested strains in the nutrient solution (Ti, DLC-Ti, and high concentrated Ag-DLC-Ti); t = 0: before incubation; t = 24 h: after incubation

Analysis of planktonic growth in the growth medium showed, in accordance to the results of surface growth, strain dependent differences. Initial bacterial concentration (t = 0) of all strains was approximately 1 × 10^5^ CFU/ml and increased in the absence of Ag after 24 h of incubation on average up to 4.5 × 10^5^ CFU/ml for *S. epidermidis*, 1.1 × 10^8^ CFU/ml for *S. aureus* and 2.4 × 10^8^ CFU/ml for *P. aeruginosa* (Fig. 4). Compared to native plates, DLC-Ti samples led to slight strain dependent changes of bacterial concentration which were only significant for *S. epidermidis*. Equally to the reduction of bacterial adherence on Ag-DLC-Ti samples a significant reduction of bacterial planktonic growth in the supernatant medium was observed for all strains in the presence of Ag.

### Antimicrobial effect of low concentrated (Ag:PVP = 1:10 and 1:20) Ag-DLC-Ti on *S. aureus* ATCC25923

**Fig. 5:**
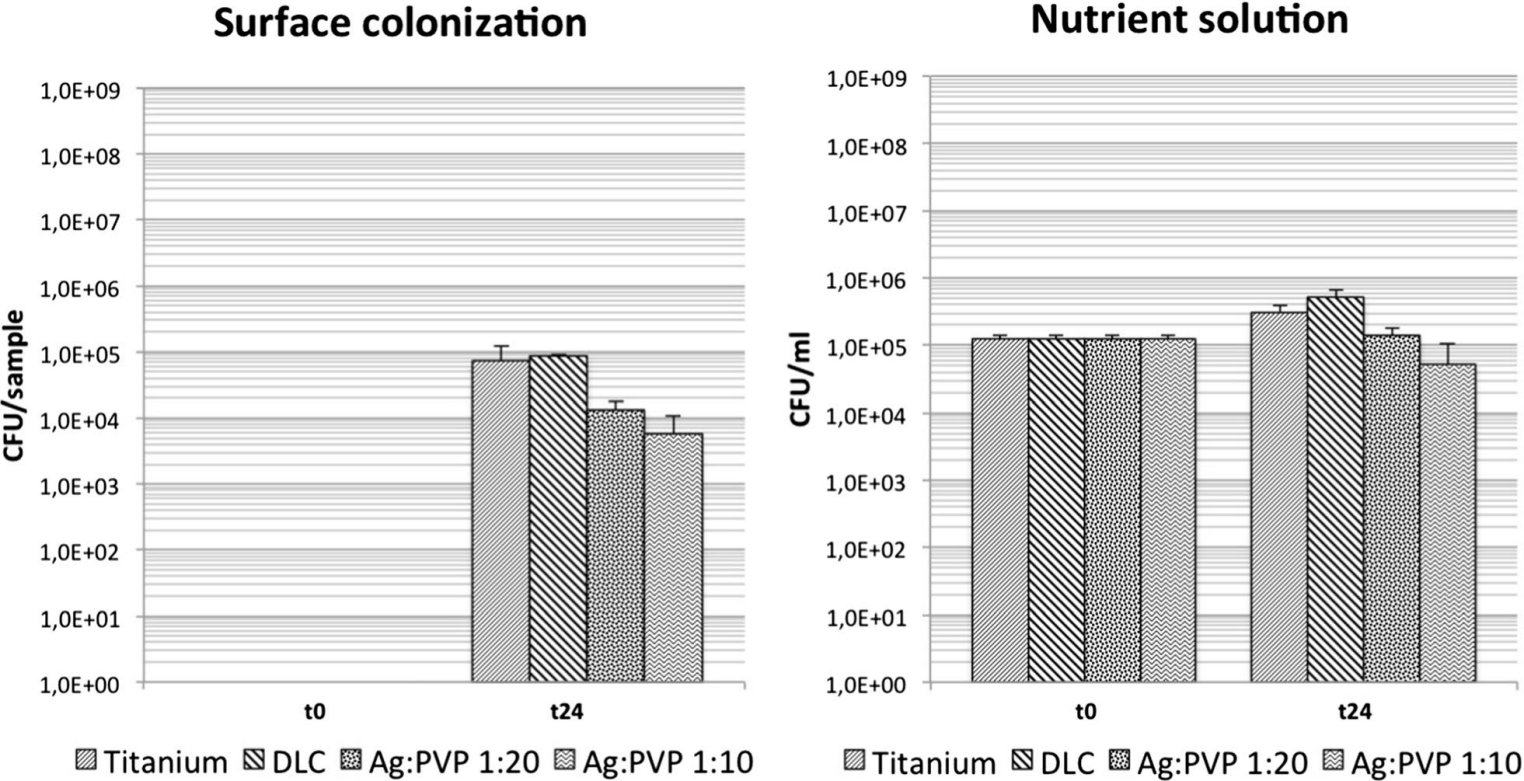
Bacterial growth of *S. aureus* on the surface and in the nutrient solution (Ti, DLC-Ti, and low concentrated Ag-DLC-Ti); t = 0: before incubation; t = 24 h: after incubation

Analysis of bacterial growth showed significantly decreased bacterial concentrations of *S. aureus* on the surface and in the growth medium for reduced Ag concentrations of Ag-DLC-Ti (Table 2; Fig. 5).

### Surface biofilm formation (*S. epidermidis* RP62a) in SEM-images

**Fig. 6:**
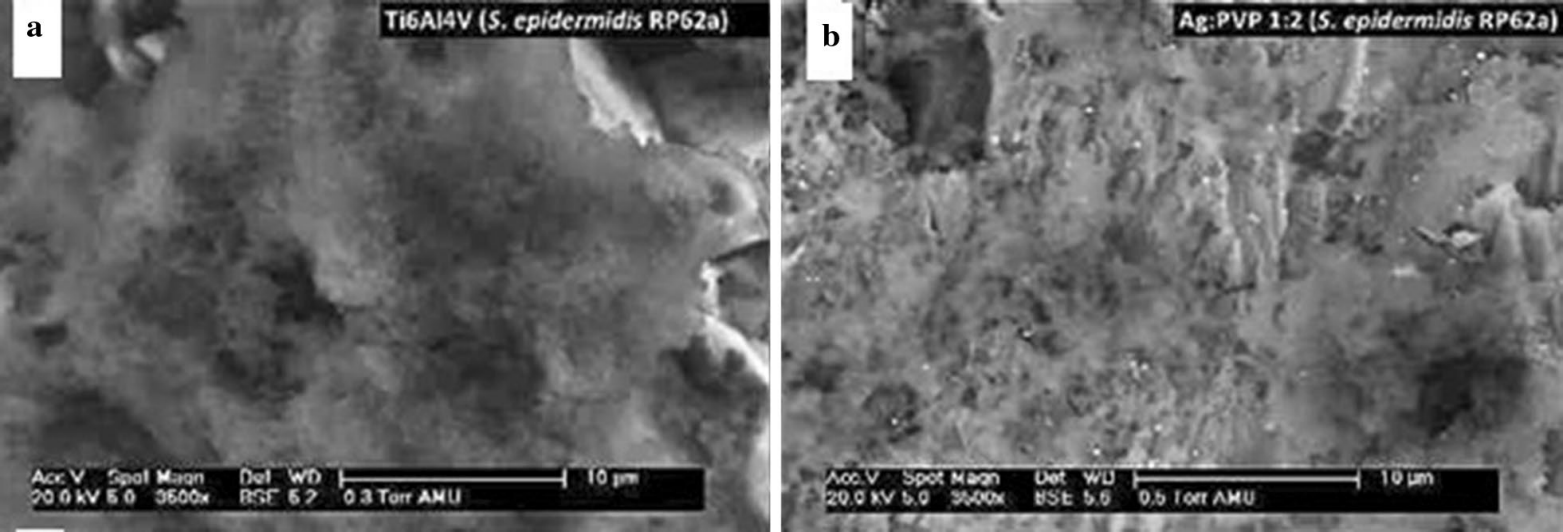
Biofilm formation on different Ti surfaces. Homogenous biofilm grade 5 after incubation with *S. epidermidis* on native Ti (**a**), reduced biofilm grade 3 on high concentrated Ag-DLC-Ti (**b**)

Biofilm formation was ubiquitous and graded type 5 on all untreated Ti and DLC-Ti samples without Ag incorporation covering the entire specimen surfaces with thick layers of *S. epidermidis*. Ag-DLC-Ti samples (Ag:PVP = 1:2) on the other hand showed biofilm inhibiting effects with at the most rare spot-like biofilm formation graded type 3 (Fig. 6a, b).

## Discussion

In the present study, the results from in vitro assays demonstrated that Ag-DLC-Ti effectively prevented bacterial adherence and biofilm formation (Table 2). In addition, we also found significant antibacterial activity in the surrounding environment of the tested samples showing release of embedded bactericidal agents from the DLC coating. In this context main properties of the tested coatings could be identified:

1. Antimicrobial effectiveness increased with higher concentrations of Ag in DLC-Ti (Ag:PVP = 1:2 vs. Ag:PVP = 1:10 and 1:20) and was still present with low concentrated Ag-DLC-Ti samples. From a physical point of view this is not surprising since high ion concentrations determine a higher rate of Ag dissolution from the nanoparticles into the surrounding medium. This “wash-out” effect of DLC surfaces on ions implanted with high concentrations has already been described in the literature in other materials (Furno et al. 2004). Several studies confirmed the bactericidal effect of Ag-DLC coatings of different materials (Soininen et al. 2011; Kwok et al. 2007; Baba et al. 2013; Katsikogianni et al. 2006; Marciano et al. 2009). However, to our best knowledge, our presented evaluation of different concentrations of Ag-DLC-Ti manufactured by dip-coating and PIII is the first described so far. In the study of Baba et al. Ag-DLC coating was achieved by a combination of magnetron sputtering and plasma source ion implantation. Different concentrations of Ag were used, but no significant differences of bactericidal effects from high and low concentrated Ag-DLC coatings were found (Baba et al. 2013). This emphasizes the importance of the manufacturing process of DLC onto the dissolution of Ag^+^ from the coating. Nevertheless, bactericidal effects can also be explained by interactions of bacteria with the surface and not only by release of silver-nanoparticles. Contradicting statements exist whether DLC coating alone exhibits bactericidal effects or not. Marciano et al. (2009) found a significant bactericidal potency of DLC, other investigators found no antibacterial effect (Soininen et al. 2011; Baba et al. 2013). In our study DLC coating alone had no significant bactericidal effect compared to untreated Ti. Similar to the findings of Soininen et al. (2011), who used the same bacterial strains, *S. epidermidis’* and *S. aureus’* adhesion on DLC coatings were slightly higher compared to uncoated Ti samples (0.2 and 0.03 log-levels; p > 0.05). Adhesion of *P. aeruginosa* on the other hand was slightly lower (0.09 log-levels; p > 0.05). This proves the statement that bactericidal effects in the present study derived mainly from the release of Ag^+^ from the coating.
2. Another main finding of our study is the different susceptibility against Ag-doped DLC coatings depending on the bacterial strain. *P. aeruginosa* showed the highest susceptibility (reduced surface growth by 5.6 log-levels; p < 0.05) followed by *S. epidermidis* (reduced surface growth by 4.4 log-levels; p < 0.05) and *S. aureus* (reduced surface growth by 2.6 log-levels; p < 0.05). In general, several studies confirmed higher bactericidal potency of Ag against Gram-negative compared to Gram-positive strains (Flores et al. 2013; Taglietti et al. 2012; Kim et al. 2007). Ag acts by binding to membranes, enzymes and nucleic acids. Consequently the respiratory chain is inhibited and therefore the aerobe metabolism of microorganisms disturbed (Gosheger et al. 2004). Bacteria are quite susceptible to Ag with only negligible possibility of intrinsic resistance (Kumar and Munstedt 2005). Nevertheless, the effect observed in the present study is not fully understood. Different cell morphology or generation time of the bacteria may only be two of several reasons for our findings (Morones et al. 2005). However, this finding is important for the future use of Ag-doped DLC surfaces since PJI involve a variety of different bacterial strains with different susceptibilities against Ag.

This study involves several limitations. Ion concentrations of Ag in the surrounding medium were not assessed. Antibacterial effects in the surrounding medium and on the sample surface could be caused or at least supported by antiadhesive surface features of DLC alone. Even though, compared to untreated Ti, no significant reduction of growth of *S. aureus* in the growth medium and *P. aeruginosa* on the surface and in the growth medium was detected for DLC-Ti, a tendency to diminished bacterial amounts of these bacteria was observed (Table 2). Another limitation is that only *S. aureus* was investigated for low concentrated Ag-DLC-Ti. It can be estimated that the bactericidal effect on *P. aeruginosa* and *S. epidermidis* would be also evident if low concentrated Ag-DLC coatings were used, since *S. aureus* was the least susceptible strain of the three in the high concentrated testing group. Additionally, no influence of Ag-DLC-Ti on osseointegration was investigated. It is known, that DLC coating of Ti can lead to increased osseointegration (Mändl et al. 2001). If this effect of DLC coatings is inhibited in the presence of Ag is unknown. Further investigations are needed in order to clear whether the concentration and duration of delivery of the released Ag^+^ of DLC coatings is sufficient to avoid implant infection in vivo and how they interact with eukaryotic cells. However, this was not the scope of this proof of principle investigation. Lastly, we did not evaluate the tribological behavior of this coating. DLC coatings of Ti exhibited good results in experimental wear studies, if Ag-DLC coatings do also is unknown (Xu and Pruitt 1999; Brizuela et al. 2002) but is the aim of further studies.

In summary, our findings show that Ag-DLC-Ti manufactured by a modified technique of dip-coating and ion implantation has considerable effects as an antibacterial coating. Thus, Ag-DLC-Ti can be considered a promising material for next generation orthopedic devices. The suitability of this coating for biomedical applications will be confirmed by wear tests and in vitro biocompatibility assessments.

## Abbreviations

Ag: silver
Ag^+^: silver ions
Ag-DLC: silver incorporated diamond-like carbon coating
Ag-DLC-Ti: silver incorporated diamond-like carbon coating on titanium
CFU: colony forming units
DLC: diamond-like carbon
DLC-Ti: diamond-like carbon coating on titanium
PIII: plasma immersion ion implantation
PJI: periprosthetic joint infections
SD: standard deviation
Ti: corundum-blasted medical titanium (TiAl6V4) alloy
